# Clinical effectiveness and safety of sirolimus in pediatric patients with complex vascular anomalies: necessitating personalized and comprehensive approaches

**DOI:** 10.3389/fped.2023.1304133

**Published:** 2023-11-16

**Authors:** Minji Kim, Kyung Taek Hong, Hyun Jin Park, Bo Kyung Kim, Jung Yoon Choi, Hyun-Young Kim, Hyoung Jin Kang

**Affiliations:** ^1^ Department of Pediatrics, Seoul National University College of Medicine, Seoul National University Children’s Hospital, Seoul, Republic of Korea; ^2^ Seoul National University Cancer Research Institute, Seoul, Republic of Korea; ^3^ Division of Pediatric Surgery, Seoul National University College of Medicine, Seoul, Republic of Korea; ^4^ Wide River Institute of Immunology, Hongcheon, Republic of Korea

**Keywords:** sirolimus, vascular anomalies, pediatrics, child, lymphatic malformation, vascular tumor

## Abstract

**Background:**

Managing complex vascular anomalies in pediatric care requires comprehensive approaches. Sirolimus, an mTOR inhibitor with immunosuppressive and anti-angiogenic properties, offers promise. We evaluated sirolimus's effectiveness and safety in pediatric patients with complex vascular anomalies at a tertiary children's hospital.

**Methods:**

Our study included 20 patients, aged 1 month to 19 years, with diverse vascular anomalies resistant to conventional therapies or located in high-risk areas precluding surgery. The evaluation of response encompassed measuring the reduction in the size of the targeted vascular or lymphatic lesions as observed on radiologic imaging, along with considering improvements reported by the patients.

**Results:**

Patients used sirolimus for a median of 2.1 years, ranging from 0.6–4.3 years. Results indicated that 60% of patients achieved complete or partial response (CR/PR), whereas 40% had stable disease (SD). Notably, no disease progression occurred. Lesion size assessment was complex, yet patients' self-reported improvements were considered. Three patients reinitiated sirolimus after discontinuation due to worsening lesions. Sirolimus treatment demonstrated good tolerability, with minor complications except for one case of Pneumocystis jiroveci pneumonia. Group comparisons based on response highlighted better outcomes in patients with vascular tumors (CR/PR group 58.0% vs. SD group 0.0%, *P* = 0.015) or localized measurable lesions (83.3% vs. 12.5%, *P* = 0.005).

**Conclusion:**

Our study underscores sirolimus's potential for treating complex vascular anomalies in pediatric patients. Challenges associated with optimal treatment duration and concurrent interventions necessitate a comprehensive approach and genetic testing to optimize outcomes.

## Introduction

Vascular anomalies, including vascular tumors and vascular or lymphatic malformations, pose a significant challenge in pediatric care due to their aggressive growth and potential complications (1). A multidisciplinary team approach is crucial, particularly for patients with inoperable or sclerotherapy- and medication-resistant vascular anomalies.

Sirolimus, a mammalian target of rapamycin (mTOR) inhibitor, has emerged as a promising therapeutic agent for refractory vascular anomalies (2). Sirolimus demonstrates potent immunosuppressive and anti-angiogenic properties, inhibiting the mTOR pathway, critical in angiogenesis and vascular endothelial cell proliferation (3). In preclinical studies, sirolimus has shown efficacy in inhibiting hemangioma growth by reducing endothelial cell proliferation, inducing apoptosis, and suppressing pro-angiogenic factors (4). Furthermore, applying sirolimus in complex vascular anomalies in pediatric patients could potentially reduce lesion size, as reported by several previous studies (3, 5–8).

However, the diversity of vascular anomaly types makes standardizing treatment challenging, and potential racial differences that warrant further research exist (9, 10). In addition, the experience of sirolimus in treating vascular anomalies among Koreans is currently limited (7). This study investigated the clinical effectiveness and potential adverse events associated with sirolimus in patients aged <19 years. These patients underwent sirolimus treatment for > 6 months at a single tertiary children's hospital in Korea. Notably, all patients presented with vascular anomalies that were either inoperable or presented challenges for surgical removal or other local therapies, including sclerotherapy. Moreover, most of these anomalies had shown limited response to previous treatments. This study primarily aimed to evaluate the treatment's impact on the disease. This assessment encompassed measuring reductions in the size of targeted vascular or lymphatic lesions, as observed through radiologic imaging, while also considering self-reported improvements from the patients.

## Methods

The data was retrospectively reviewed from 20 patients with vascular tumor, venous malformation (VM), and lymphatic malformation (LM) treated with sirolimus between April 2019 and August 2023 at Seoul National Children's Hospital. Patients’ sex, diagnosis, lesion location, previous treatment history, age at initiation of sirolimus, duration of sirolimus, treatment response, and adverse events were reviewed from the medical records.

In this study, vascular anomalies were diagnosed through clinical assessment and confirmed using imaging techniques such as magnetic resonance imaging, ultrasonography, or computed tomography scans. We used nomenclature based on the disease classification system according to the ISSVA (International Society for the Study of Vascular Anomaly) (10, 11).

Between April 2019 and August 2023, 23 patients were treated with sirolimus for diagnoses including LM, VM, kaposiform hemangioendothelioma (KHE), congenital hemangioma (CH), and Gorham-Stout disease (GSD). Notably, most patients did not respond to previous treatments such as other medications (propranolol, prednisolone), interventions (sclerotherapy or embolization), or surgery. Similarly, some patients had lesions in risky areas, making them unsuitable candidates for surgery. The study specifically focused on children and adolescents aged ≤19. The inclusion criterion was undergoing sirolimus treatment for a minimum duration of 6 months. Consequently, the analysis did not include patients who used sirolimus for <6 months. In addition, patients who were transferred to other institutions were excluded from the study population. The sirolimus treatment typically began with an initial oral dose of 0.8mg/m^2^, administered twice daily. The desired target trough level for sirolimus in the bloodstream ranged from 5 to 15 ng/ml.

The optimal measure of disease response in patients with complex vascular anomalies has not been established. To provide a more comprehensive assessment, we employed two primary criteria to assess disease response, both of which relied on imaging results and changes in patients' symptoms in our study (7, 8). A “Complete Response” meant there was no evidence of disease on imaging, no organ dysfunction due to the disease, and a return to a normal quality of life. “Partial Response” (PR) was when the size of the vascular lesion reduced by more than 20% on imaging, or there was at least a one-grade improvement in organ dysfunction. “Progressive Disease” (PD) was when the vascular lesion increased by more than 20% on imaging or there was a one-grade worsening in organ dysfunction. If none of these criteria were met, the patient was considered to have “Stable Disease” (SD). Additionally, we also used a “Good/Intermediate/Poor Response” system. “Good Response” meant an improvement of over 70% on imaging or the absence of a visible lesion. “Intermediate Response” was an improvement between 30% and 70% on imaging or self-reported improvement of the lesion. “Poor Response” was when the improvement on imaging was less than 30% or the disease remained stable, or there was self-reported worsening of the lesion (Table 1).

**Table 1:** Criteria of disease response.

A. Complete or Partial Response/Stable or Progressive disease response system (8)	
Disease response will be established by changed in at least 1 parameter, coded by using the following criteria - Response by imaging - Assessment of other clinical measures (quality of life) - Clinical criteria and functional impairment Response was established by changes in at least 1 of these parameters	
Complete Remission	No evidence of disease on radiologic imaging and
	No evidence of organ dysfunction due to disease and
	Normalization of quality of life criteria
Partial Response	>20% reduction in size of target vascular lesion evident on radiologic imaging or
	Improvement in target organ dysfunction by at least 1 grade or
	Improvement of self-report PedsQL by >4.4 or proxy-report PedsQL by >4.5 compared with baseline; FACT-G by >3.99
Progressive Disease	>20% increase in size of target vascular lesion evident on radiologic imaging or
	Worsening in target organ dysfunction by at least 1 grade or
	Worsening of self-report PedsQL by >4.4 or proxy-report PedsQL by >4.5 compared with baseline; FACT-G by >3.99
Stable Disease	None of the above
B. Good/Intermediate/Poor response system (7)	
Response	Description
Good	Improvement in radiologic imaging findings of >70% or remnant lesion in radiologic imaging, but no gross lesion identified
Intermediate	Improvement in radiologic imaging findings of ≤70% and >30%
	Or self-reported improvement of gross lesion
Poor	Improvement in radiologic imaging findings of <30%, or stable disease status
	Or self-reported worsening of gross lesion

At our institution, we regularly hold a comprehensive meeting involving a diverse team of experts, including pediatric surgeons, plastic and orthopedic surgeons, radiologists, dermatologists, pathologists, pediatric hemato-oncologists, and other specialized professionals. This collaborative approach is crucial for deliberating and selecting the most appropriate treatment options for patients with complex vascular anomalies. Within this team, pediatric radiologists play a vital role in evaluating these anomalies and precisely measuring their size to inform treatment decisions. Ultimately, the attending clinician, who evaluates both the patient's symptoms and radiological changes, makes the final determination of clinical responses.

Data analysis was conducted using the statistical software SPSS 23.0 (IBM, Armonk, NY). We performed chi-square tests and Fisher's exact tests for nominal variables. However, we compared the mean values of the PR/Intermediate and SD/Poor groups for quantitative variables and conducted a t-test. This study was approved by the Institutional Review Board of Seoul National University Hospital, and the requirement for consent was waived (H-2308-136-1459).

## Results

### Patient population

Three of the initial 23 patients did not meet the inclusion criteria and were therefore excluded. Consequently, 20 patients were enrolled in the study. One patient, aged 48, who presented with extensive venous malformation on the right arm was excluded due to not meeting the age criteria specified in the study despite reporting symptomatic improvement. The remaining patients were excluded due to transferring to another hospital before evaluation and using sirolimus for <6 months, respectively.

A group of 20 patients was selected for the study. Fourteen were male, and the median age for starting sirolimus treatment was 6.9 years old, ranging from 1 month to 19 years. Among the cases, 35% were classified as LM, 25% as VM, 30% as KHE, and 10% as GSD. The head and neck were the areas most frequently affected by the lesions, followed by the lower extremities, back, chest, and upper extremities. Patients used sirolimus for a median of 2.1 years, with durations ranging from 0.6–4.3 years. Among the 20 patients we studied, 15 (75%) had previously undergone surgical excision, embolization, sclerotherapy, or medical treatment (propranolol or prednisolone). Eleven out of the 15 patients showed a poor response to pre-sirolimus medical or surgical treatments. The remaining patients had lesions located in challenging areas like the neck or orbit area, unsuitable for surgery and procedures.

### Response and reinitiation of sirolimus

**Figure 1:**
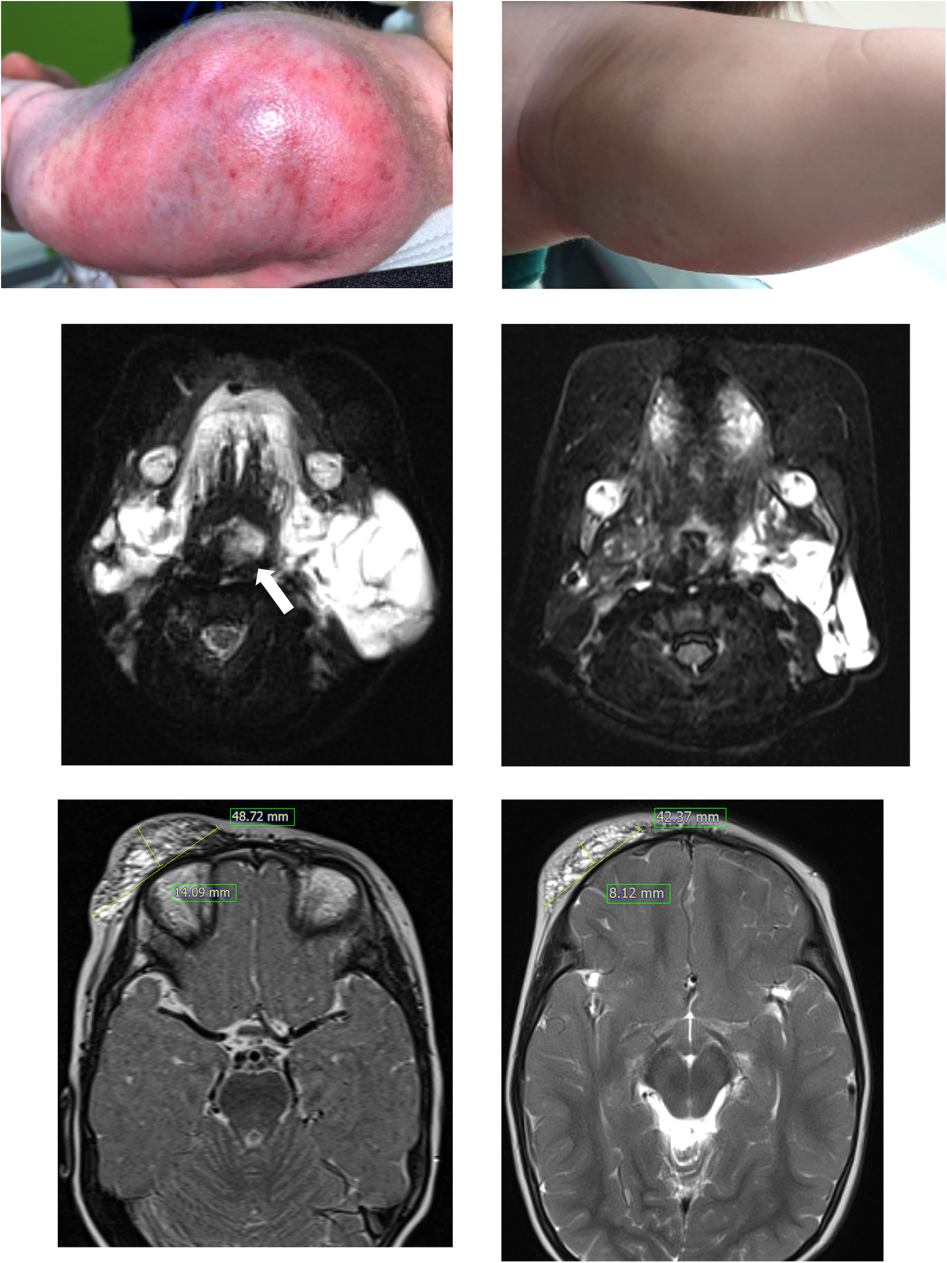
Patient 1 had a large kaposiform hemangioendothelioma on her left upper arm (**A**), and the mass showed improvement after nine months of sirolimus administration (**B**). In this T2-weighted MRI, patient 3, who presented with a large cystic mass on the lower left neck and around the airway (indicated by the arrow) at birth (**C**), experienced relief from airway compression after 5 months of sirolimus treatment (**D**). Patient 4 showed a protruding mass suspected to be a lymphatic malformation on the right forehead and eyelid (**E**), and a T2-weighted MRI was obtained after 6 months of sirolimus treatment (**F**).

According to Table 2, one patient was evaluated to have achieved complete remission and a good response, with all lesions having disappeared. Of the 20 patients, 11 (55%) showed partial and intermediate responses, and eight (40%) showed stable disease and poor responses. There was no case of progressive disease (Figures 1A–E). Unfortunately, measuring the exact size of lesions was challenging due to the extensive and/or diffuse shape. Therefore, self-reported symptom changes were also considered important indicators of the sirolimus response.

**Table 2:** The summary of patient characteristics and treatment response.

Patient number	Diagnose	Age at initiation of sirolimus(years)	sex	Location of lesions	Previous treatment (duration) / Disease response of previous treatment	Duration of sirolimus(years)	Additional treatment with sirolimus	Target mass size change (% of compared to the previous lesion size)	Self-reported changes in symptoms	Disease Response (Ref 8 / Ref 7)	Adverse Effects (CTCAE Grade)	Current status
1	KHE	0.2	F	Lt arm	Pred (3wk), PPL (2wk) / Poor	3.5	N/A	8.6 × 4.3 × 6.8 cm → 2.2 × 2.0 × 6.9 cm (12.1%)	The swelling, redness and heat sensation on the lesion improved.	PR/ Intermediate	Pneumocystis Jiroveci infection (3)	Reinitiation after D/C^a^, Ongoing
2	LM	6.1	F	Lung, pericardium, abdomen, spleen	IVIG #1, Pred,(7mo) Embolization #2 / Poor	3.4	N/A	Unmeasurable due to extensive and diffuse lesions	No reported specific change.	SD/ Poor	dyspepsia (1), fatigue (2)	Reinitiation after D/C^b^, Ongoing
3	LM	0.1	M	Neck	None	3.3	Sclerotherapy #1	4.5 × 3.0 × 2.7 cm → 6.2 × 1.9 × 3.2 cm (103.4%)	Initial respiratory difficulty improved; otherwise, stationary.	SD/ Poor	recurrent upper respiratory infection (2)	D/C
4	LM	1.1	M	Forehead, orbit	None	3.3	Sclerotherapy #3	1.4 × 4.9 × 3.3 cm → 0.8 × 4.5 × 2.8 cm (44.5%)	Right eye opened wider.	PR/ Intermediate	none	ongoing
5	LM	2.4	F	Neck	Sclerotherapy #3 / Poor	2.8	Excision of lymphangioma #1	5.8 × 5.5 × 3.7 cm → 2.7 × 5.0 × 3.1 cm (35.5%)	No reported specific change.	PR/ Intermediate	recurrent upper respiratory infection (2)	Reinitiation after D/C^c^ Ongoing
6	LM	16.1	F	Buttock	Mass excision #1 / Intermediate	2.7	N/A	9.2 × 4.4 × 10.0 cm → 8.6 × 3.3 × 9.2 cm (64.5%)	Buttock pain improved	PR/ Intermediate	none	ongoing
7	LM	11.6	M	Buttock, thigh, perianal area	mass excision #2, Electrocauterization #2 / Intermediate	0.6	N/A	Unmeasurable due to extensive and diffuse lesions	No reported specific change.	SD/ Poor	Hyperglycemia (1)	D/C
8	KHE	0.2	M	Rt cheek	Pred (1 mo), PPL (1 mo) / Poor	2.2	Pred (6m), VCR (2m)	6.1 × 4.0 × 4.4 cm → 3.3 × 2.3 × 2.4 cm (17.0%)	The swelling, redness and heat sensation on the lesion improved.	PR/ Intermediate	hypercholesterolemia (1)	D/C
9	LM	13.8	M	Neck, tongue	Excision#2, Sclerotherapy #2, glossoplasty #1 / Poor	1.1	N/A	Unmeasurable due to diffuse lesions	Tongue discomfort and bleeding did not change.	SD/ Poor	oral mucositis (2)	D/C
10	KHE	1.1	M	scapula and acromion	Pred (4 mo) / Intermediate	4.3	Pred (5mo)	2.5 × 1.0 × 2.3 cm → 1.9 × 0.9 × 1.4 cm → none		CR/ Good	herpatic gingivostomatitis (2)	D/C
11	CH	0.4	M	Rt. Back, flank	Pred (2 mo), PPL (2 mo) / Poor	1	N/A	6.0 × 5.0 cm → 5.0 × 5.0 cm^d^	The swelling, redness and heat sensation on the lesion improved.	PR/ Intermediate	none	ongoing
12	VM	2	F	Rt. Check, lip	Laser treatment, topical PPL / Poor	1	N/A	Unmeasurable due to diffuse lesions	No reported specific change.	SD/ Poor	Oral mucositis (2)	D/C
13	KHE	4	M	cheek mass	Pred (1 mo), PPL (1 mo) / Intermediate	2.1	N/A	2.7 × 1.7 × 2.0 cm → 2.3 × 1.2 × 1.9 cm (57.1%)	The swelling, redness and heat sensation on the lesion improved.	PR/ Intermediate	none	ongoing
14	VM	18.8	F	Rt. neck, mandible, mouth floor, oropharynx	None	1	N/A	Unmeasurable due to extensive and diffuse lesions	Oral pain improved	PR/ Intermediate	oral mucositis (2)	ongoing
15	KHE	12.8	M	Thigh, Foot	Curettage #1 / Poor.	1.1	N/A	6.0 × 3.0 × 10.0 → 3.9 × 3.6 × 8.8 cm (68.7%)	Pain improved	PR/ Intermediate	none	ongoing
16	KHE	0.2	M	neck mass	Pred (4 mo), PPL (3 mo) / Poor	1.4	N/A	6.0 × 3.6 × 2.8 cm → 4.0 × 3.2 × 2.7 cm (57.1%)	No reported specific change.	PR/ Intermediate	none	ongoing
17	VM	9.9	M	Rt. Leg, Hip, Foot	None	2.1	Sclerotherapy #4	Unmeasurable due to extensive and diffuse lesions	The overall pain and heaviness in the right leg have improved.	PR/ Intermediate	none	ongoing
18	GSD	14	M	Pelvic cavity, Abdomen, Rt. Leg to Axilla	Thoracic duct embolization #1 / Poor	3.6	Pamidronate, Lymphovenous anastomosis, radiotherapy	Unmeasurable due to extensive and diffuse lesions	Pleural effusion and lymphedema waxed and waned.	SD/ Poor	none	ongoing
19	GSD	8.6	M	Rt iliac bone, lumbosacral vertebral bodies, inguinal	None	1	N/A	Unmeasurable due to extensive and diffuse lesions	Pelvic pain waxed and waned	SD/ Poor	non-cardiac chest pain (1), sinus tachycardia (1)	ongoing
20	VM	14.5	M	Chest, shoulder	Laser treatment, topical PPL / Poor	0.6	N/A	Unmeasurable due to diffuse skin lesions	The overall red color has lightened, but the extent of the lesion remains similar	SD/ Poor	oral mucositis (1)	ongoing

CH, Congenital Hemangioma; CR, Complete Response; GSD, Gorham-Stout Disease; D/C, discontinued; IVIG, Intravenous immunoglobulin; KHE, Kaposiform Hemangioendothelioma; LM, Lymphatic Malformation; N/A, not applicable; PD, Progressive Disease; PR, Partial Response; Pred, Prednisolone; PPL, propranolol; SD, Stable Disease; VCR, Vincristine; VM, Venous Malformation.

^a^ Restarted sirolimus after a 9-month period off due to an increase in the size of the lesion.

^b^ Restarted sirolimus after 6-month period off due to relapse of pleural effusion.

^c^ Restarted sirolimus after 3-month period off due to an increase in the size of the lesion.

^d^ Evaluated by physical exam. Depth could not be measured, however the previously swollen lesion has significantly flattened.

Three patients (two with LM and one with KHE) reinitiated sirolimus after discontinuation due to worsening lesions. Patient 1 showed marked improvement in swelling and heat sensation of the lesion and discontinued the medication due to lesion stability and inactivity for 1 year. However, the lesion worsened after 9 months off the medication, with swelling and heat sensation reoccurring, which improved after reinitiating sirolimus. Patient 2 had an extensive LM affecting major lymphatic vessels, including the thorax. Sirolimus was discontinued due to prolonged stable status and possible adverse effects, such as general weakness and gastrointestinal problems. However, the patient experienced severe pleural effusion and dyspnea after 6 months of discontinuation. Sirolimus was reinitiated in combination with lymphatic embolization. Patient 5 had a large LM on the neck, the size of which decreased with sirolimus treatment. Subsequently, mass excision was performed. However, the mass could not be completely excised, and recurrent lymphatic fluid collection led to the reinitiation of sirolimus. Six patients (30%) discontinued sirolimus. Among them, four patients had a poor response to sirolimus, leading to medication discontinuation (median duration of 1.1 years). In two patients, the lesions improved due to the effects of sirolimus, and as the lesions were no longer active, the medication was discontinued.

### Adverse events

Regarding adverse events, sirolimus treatment was generally tolerable, and 12 patients (60%) experienced potential complications, including oral mucositis, recurrent upper respiratory infections, hyperglycemia, and hypercholesterolemia. All of these complications were graded at ≤2, except for one case of Pneumocystis jiroveci infection, which required 2 weeks of intravenous antibiotic treatment (Table 2). In response to this event, we initiated sulfamethoxazole/trimethoprim prophylaxis, specifically targeting patients who were either concurrently using sirolimus and prednisolone or had a history of recurrent infections (7 out of 20 patients). Following the introduction of this prophylactic measure, there were no instances of Pneumocystis jiroveci infections.

### Comparison between the CR/PR and SD groups

When we compared the CR/PR group (12 patients) to the SD group (eight patients), we found that a significantly larger portion of patients in the CR/PR group were diagnosed with vascular tumors compared to the SD group (58.0% vs. 0.0%, *P* = 0.015). Specifically, the CR/PR group had a notably higher number of measurable lesions than the SD group (83.3% vs. 12.5%, *P* = 0.005) (Table 3). No statistically significant associations were discerned between the two groups concerning variables including age at sirolimus initiation, gender, lesion location, duration of sirolimus use, or mean trough level. Additionally, there were no differences in the proportion of patients achieving CR/PR between the two groups with and without previous treatment; both groups had a CR/PR proportion of 60%. Nonetheless, it is significant that all three patients exhibiting extensively dispersed lesions across their bodies were categorized within the SD group (Table 2).

**Table 3:** Comparison between groups according to treatment response.

Variables	CR/PR group (*n* = 12)	SD group (*n* = 8)	*p* value
Age (year)	1.8 (0.2–18.8)	5.1 (0.1–18.8)	0.285
Sex			1.000
Male	8 (66.7%)	6 (75.0%)	
Female	4 (33.3%)	2 (25.0%)	
Diagnosis			0.015
Vascular tumor	7 (58.3%)	0 (0.0%)	
VM or LM	5 (41.7%)	8 (100%)	
Location			0.083
Head or Neck	6 (50.0%)	3 (37.5%)	
Extremities	3 (25.0%)	0 (0.0%)	
Trunk	3 (25.0%)	2 (25.0%)	
Extensive lesions	0 (0.0%)	3 (37.5%)	
Measurable lesions (yes)	10 (83.3%)	1 (12.5%)	0.005
Duration of sirolimus treatment (year)	2.2 (1.0–4.3)	1.1 (0.6–3.6)	0.399
Mean serum trough level of sirolimus (ng/mL)	7.9 (3.6–10.3)	7.0 (6.0–14.0)	0.492

CR, Complete Response; LM, Lymphatic Malformation; PR, Partial Response; SD, Stable Disease; VM, Venous Malformation.

## Discussion

This study conducted a retrospective analysis of the clinical effectiveness of sirolimus in pediatric patients with complex vascular anomalies, revealing a 60% rate of CR or PR. This rate is lower than the response rates reported in previous studies (2, 3, 6–8). The presence of selection bias in retrospective studies and potential ethnic differences should be considered. Furthermore, it is essential to acknowledge the challenge of comparing responses to vascular anomalies using standardized assessments, given the significant variation in treatment response evaluations across different studies. In our study, although 40% of patients were categorized as SD or poor responders based on the criteria, most patients in the SD group continued sirolimus treatment due to lesion stability. Patient 3, who had LM around the neck since birth and was classified into the SD group, experienced relief from respiratory distress symptoms during infancy by initiating sirolimus treatment at 1 month. This enabled the avoidance of emergent surgical intervention. Sirolimus treatment served as a bridge, allowing the patient to undergo concurrent sclerotherapy safely at the age of three (Figures 1C,D).

Unfortunately, a definitive consensus regarding the optimal duration of sirolimus treatment was not established. Given the challenging nature of treating complex vascular anomalies, the continuation of sirolimus treatment often depended on clinical improvements and the patient's tolerance to the medication. Notably, three patients (two with PR and one with SD) in our study resumed sirolimus treatment after discontinuation. One patient, who did not meet the response criteria, ceased sirolimus treatment based on the choice of the patient's guardian. However, after a 6-month discontinuation, this patient developed significant pleural effusion and hemothorax related to lymphatic malformation in the thorax. Sirolimus was reinitiated alongside additional lymphatic embolization, resulting in the overall stabilization of lymphatic malformation until the last follow-up. This observation implies that even within the SD patient group, sirolimus might exhibit clinical utility by partially inhibiting disease progression. It will be essential to personalize the timing of drug administration for individual patients through meticulous clinical monitoring.

Careful monitoring of adverse events is crucial when administering sirolimus. Cases of potential adverse effects, such as Pneumocystis jirovecii pneumonia infection, have been reported; however, this is rare. In addition, hyperlipidemia and hyperglycemia have been observed, underscoring the importance of frequent lipid and blood glucose level assessments. As the benefits and risks of sirolimus use require delicate balancing, an informed evaluation is necessary to determine the most suitable treatment duration.

In our study, patients with measurable local lesions or vascular tumors, compared with those with VM or LM, exhibited more favorable treatment responses. Age and the mean serum trough level of sirolimus were not associated with treatment outcomes. Patients with more severe and extensive vascular anomalies tended to respond less effectively to sirolimus treatment. Considering the potential diversity of therapeutic targets for vascular anomalies (12), these patients should undergo molecular analysis and could be regarded as candidates for alternative medications, such as alpelisib (13) or trametinib (14). These medications have also shown promising responses in patients who did not respond well to sirolimus. However, longer follow-up to assess these new medications’ sustained response and long-term adverse events is essential, particularly for pediatric patients.

Due to the heterogeneity of diagnoses, a limited number of patients, and the retrospective nature of the analysis, the interpretation of our study is constrained. Including five patients who received supplementary sirolimus treatment for improved disease control posed challenges in accurately assessing the effectiveness of sirolimus alone. Nonetheless, our study offers valuable real-world data, highlighting the necessity for comprehensive treatment. Considering the functional and cosmetic concerns in pediatric patients with complex vascular anomalies, a multidisciplinary team approach becomes imperative for enhancing disease control and promoting normal growth and development.

In conclusion, our study demonstrated promising outcomes and a well-tolerated safety profile of sirolimus in pediatric patients with complex vascular anomalies at a tertiary children's hospital in Korea. Nevertheless, specific unresolved issues pertain to treatment duration and the timing of concurrent interventions. A multidisciplinary team approach and genetic testing of the lesions would be imperative to enhance long-term outcomes.

## Data Availability

The raw data supporting the conclusions of this article will be made available by the authors, without undue reservation.
